# Disrupted individual‐level morphological brain network in spinal muscular atrophy types 2 and 3

**DOI:** 10.1111/cns.14804

**Published:** 2024-06-17

**Authors:** Yufen Li, Huirong Nie, Pei Xiang, Wanqing Shen, Mengzhen Yan, Cui Yan, Shu Su, Long Qian, Yujian Liang, Wen Tang, Zhiyun Yang, Yijuan Li, Yingqian Chen

**Affiliations:** ^1^ Department of Radiology The First Affiliated Hospital of Sun Yat‐sen University Guangzhou China; ^2^ Department of Interventional Oncology The First Affiliated Hospital of Sun Yat‐sen University Guangzhou China; ^3^ Department of Pediatric Intensive Care Unit The First Affiliated Hospital of Sun Yat‐sen University Guangzhou China; ^4^ Department of Biomedical Engineering, College of Engineering Peking University Beijing China

**Keywords:** graph theory, morphological brain network, spinal muscular atrophy, structural magnetic resonance imaging

## Abstract

**Background and Objective:**

Spinal muscular atrophy (SMA) is one of the most common monogenic neuromuscular diseases, and the pathogenesis mechanisms, especially the brain network topological properties, remain unknown. This study aimed to use individual‐level morphological brain network analysis to explore the brain neural network mechanisms in SMA.

**Methods:**

Individual‐level gray matter (GM) networks were constructed by estimating the interregional similarity of GM volume distribution using both Kullback–Leibler divergence‐based similarity (KLDs) and Jesen‐Shannon divergence‐based similarity (JSDs) measurements based on Automated Anatomical Labeling 116 and Hammersmith 83 atlases for 38 individuals with SMA types 2 and 3 and 38 age‐ and sex‐matched healthy controls (HCs). The topological properties were analyzed by the graph theory approach and compared between groups by a nonparametric permutation test. Additionally, correlation analysis was used to assess the associations between altered topological metrics and clinical characteristics.

**Results:**

Compared with HCs, although global network topology remained preserved in individuals with SMA, brain regions with altered nodal properties mainly involved the right olfactory gyrus, right insula, bilateral parahippocampal gyrus, right amygdala, right thalamus, left superior temporal gyrus, left cerebellar lobule IV–V, bilateral cerebellar lobule VI, right cerebellar lobule VII, and vermis VII and IX. Further correlation analysis showed that the nodal degree of the right cerebellar lobule VII was positively correlated with the disease duration, and the right amygdala was negatively correlated with the Hammersmith Functional Motor Scale Expanded (HFMSE) scores.

**Conclusions:**

Our findings demonstrated that topological reorganization may prioritize global properties over nodal properties, and disrupted topological properties in the cortical–limbic‐cerebellum circuit in SMA may help to further understand the network pathogenesis underlying SMA.

## Graphical abstract

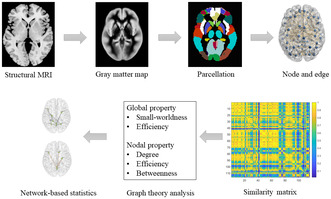

## 1 INTRODUCTION

Spinal muscular atrophy (SMA) is an autosomal recessive disease caused by the deletion or mutation of the survival motor neuron 1(SMN1) gene and the resulting insufficient expression of the SMN protein.^1^, ^2^ The core clinical manifestation is progressive, symmetric, proximal muscle weakness, and atrophy. To date, the main focus has been on the motor unit in SMA, including motor neuron loss in the spinal cord,^3^ neuromuscular junction abnormality,^4^ and progressive atrophic muscle,^5^ but the involvement of brain neural circuits underlying SMA is not well understood.

Growing evidence from clinical, neuropathological, and neuroimaging studies has shown that widespread abnormalities in the central nervous system extend well beyond the spinal cord in SMA. Post‐mortem studies^6^, ^7^ detected extensive abnormalities in the cerebral cortex, basal ganglia, thalamus, pigmented nuclei, brainstem, and cerebellum in SMA. Moreover, mouse models of SMA^8^ found that morphological changes were more obvious in brain regions with higher basal expression of SMN protein, such as the hippocampus. More recently, neuroimaging studies using voxel‐based morphological (VBM) analysis of magnetic resonance imaging (MRI)^9^, ^10^ found decreased cerebellar volume and increased motor cortex density in SMA. However, as the human brain is a complex network with highly interconnected regions, previous studies only showed extensive local morphological changes, and inter‐regional relations of local brain morphology have not been investigated in SMA.

Recently, morphological network analysis has gained increasing attention to investigate brain network changes. Population‐based morphological covariance network analysis was often used in previous studies by estimating the covariance between averaged regional morphological measures across a cohort of participants.^11^, ^12^ However, only one morphological network can be obtained based on this group‐level method, limiting the ability to investigate individual variability. Fortunately, these shortcomings can be complemented by the newly proposed individual‐level morphological similarity network method by using Kullback–Leibler divergence‐based similarity (KLDs) measurement.^13^, ^14^ This method has been widely used in many diseases, including attention‐deficit/hyperactivity disorder (ADHD),^15^ autism spectrum disorder (ASD),^16^ and spinocerebellar ataxia (SCA).^17^ To the best of our knowledge, the use of individual‐level morphological network analysis to study the brain network changes in SMA has not been reported.

Traditionally, based on the age of onset and achieved maximum motor ability, SMA is divided into three main types (types 1–3) and two less common types (the most severe type 0 for prenatal onset and the mildest type 4 for adult‐onset).^18^ Within the three main types, SMA type 1 includes infants who are unable to sit alone for the first 6 months of life, and disease progression often worsens rapidly in this subtype. In contrast, SMA types 2 and 3 are defined as late‐onset and are characterized by moderate (type 2) and relatively mild (type 3) progression. Specifically, SMA type 2 has an age of onset between 6 and 18 months and has the ability to sit but never walk independently; SMA type 3 has the onset between 18 months and 18 years with the preserved acquisition of ambulation. Besides, SMA type 0, as the most severe congenital form, was often considered to be a multiorgan disease by a previous study,^19^ with not only neuromuscular manifestations but also cardiac defect, dysautonomia, arthrogryposis, vascular necrosis, and cerebral involvement. In contrast to this, SMA type 4 with an age of onset beyond 18 years often has a normal life expectancy with slowly progressive muscle weakness.^20^ However, the results of previous studies varied partly due to the inclusion of different subgroups of SMA ranging from infants to adults.^21^ Considering sufficient SMN protein expression is crucial for the development of the central nervous system,^2^, ^22^ a higher vulnerability to SMN deficiency was expected to have a greater impact on the developmental brain. Thus, our study mainly focused on brain involvement in a broad population of SMA type 2 and 3 patients, including children and adolescents with varying states of motor dysfunction, to avoid the potential effect of hypoxic/ischemic encephalopathy on the brain structure of SMA type 1.^6^

Hence, we aimed to construct an individual‐level gray matter (GM) network by using two measurements, the KLDs and its variant Jesen–Shannon divergence‐based similarity (JSDs) and two different brain parcellation schemes, and to further investigate the topological characteristics of the GM network in individuals with SMA types 2 and 3, which encompassed a wide range of disease severity and duration. We expected to find specific network change patterns involved in SMA types 2 and 3, particularly in the cerebellar, basal ganglia, and limbic networks.

## 2 MATERIALS AND METHODS

### 2.1 Subjects

All individuals with SMA included in our study were recruited from the pediatrics and neurology departments of the First Affiliated Hospital of Sun Yat‐Sen University. Healthy controls (HCs) were recruited via open advertisement and evaluated by an experienced pediatrician to rule out any other neurologic disease. Each subject signed an informed consent form approved by the ethics committee of the First Affiliated Hospital of Sun Yat‐Sen University. The inclusion criteria for patients were as follows: (1) the individuals with SMA confirmed the diagnosis of SMA based on the gene testing; (2) according to the current consensus criteria,^23^ the individuals with SMA types 2 and 3 had an onset age between 6 months and 18 years, and SMA with prenatal or adult‐onset were not included (to rule out the possible effect of hypoxic/ischemic encephalopathy in SMA types 0 and 1); (3) the individuals with SMA were not treated with any anti‐SMA medications (i.e., nusinersen, onasemnogene abeparvovec and risdiplam) before the baseline MRI scan (only the baseline MRI scan data were used in this study); (4) the subjects were right‐handed; (5) the individuals with SMA were without head injury or any other CNS pathology; (6) MRI data were evaluated with two experienced radiologists to exclude data with obvious head movement and image artifact. For the control group, all HCs with no history of head injury, no family history of motor system disease or any other neurological disorder, and no contradiction to MRI were recruited. Meanwhile, the HCs were matched for age, gender, and right‐handedness. Finally, our study included 38 (24 male, 14 female) individuals with SMA and 38 age‐ and sex‐matched HCs.

Motor function in individuals with SMA was evaluated by the Hammersmith Functional Motor Scale Expanded (HFMSE) (ranging from 0 to 66).^24^ HFMSE consisted of 33 items using a three‐point (0–2) scale, with higher scores indicating better motor function.

### 2.2 MRI acquisition and data preprocessing

All images were acquired on a 3.0T scanner (SIGNA Pioneer GE Healthcare, WI, USA) equipped with a 32‐channel head coil. During scanning, all participants fixed their heads with foam padding to minimize head motion and wore earplugs to reduce scanning noise. Three‐dimensional high‐resolution structural T1‐weighted data were acquired using the fast spoiled gradient recalled echo (FSPGR) sequence with the following parameters: TR = 7.5 ms, TE = 3.1 ms, flip angle = 12°, slice thickness /gap = 1.0/0 mm, slice number = 188, FOV = 256 × 256 mm, and matrix = 256 × 256. T2‐weighted FLAIR images were also acquired to rule out gross cranial lesions.

Data preprocessing was conducted using the CAT12 toolbox (https://neuro‐jena.github.io/cat12/) implemented in SPM12 (http://www.fil.ion.ucl.ac.uk/spm/software/spm12/). Briefly, the main preprocessing was composed of four sections: segmentation, normalization, modulation, and smoothing. All T1 images were first segmented into gray matter (GM), white matter (WM), and cerebrospinal fluid (CSF) by using unified and refined segmentation, in which customized tissue probability maps (TMPs) created by the Template‐O‐Matic toolbox (https://neuro‐jena.github.io/software/tom) for our pediatric data were used. Then, the GM images were normalized to the study‐specific template in MNI space using the Diffeomorphic Anatomical Registration Through Exponential Lie Algebra (DARTEL) approach. To correct for volume changes due to spatial normalization, the GM images were modulated by the Jacobian determinants to preserve the original GM volume. Finally, all GM images were smoothed with a 6 mm full‐width at half‐maximum (FWHM) Gaussian kernel.

### 2.3 Construction of an individual morphological brain network

We constructed the individual morphological brain network with nodes denoting brain regions and edges denoting the interregional similarity of GM volume distribution. Particularly, previous studies have emphasized the important role of the cerebellum in SMA. For this reason, the most widely used atlas – the Automated Anatomical Labeling 116 (AAL116) atlas^25^ (consisting of 90 cerebral regions and 26 cerebellar regions), was used to define the nodes in our study. And we adopted symmetric Kullback–Leibler divergence‐based similarity (KLDs) measurement to estimate interregional morphological similarity.^14^ More specifically, the GM volume of 116 regions was extracted, and probability density functions of these 116 values were obtained using kernel density estimation with automatic estimated bandwidths for each subject. The obtained probability density functions were then further calculated as probability distribution functions (PDFs). Subsequently, a 116 × 116 similarity matrix was constructed by computing KLDs between PDFs of each pair of regions.

### 2.4 Network metrics

The global and nodal network metrics were analyzed with GRETNA software (http://www.nitrc.org/projects/gretna). The global metrics included: (1) small‐world parameters: clustering coefficient (*C*_p_), characteristic path length (*L*_p_), normalized clustering coefficient (*γ*), normalized characteristic path length (*λ*), and small‐world (*σ*); (2) network efficiency parameters: global efficiency (Eg) and local efficiency (Eloc). The nodal metrics included nodal degree, nodal efficiency, and nodal betweenness.

The sparsity threshold (S) was defined as the ratio of the existing number of edges to the maximum possible number of edges. For each metric, instead of selecting a single threshold, a wide range of thresholds were adopted to ensure the small‐world index of the network was >1.0 and to minimize the spurious edges of the network.^26^ The threshold of our study ranged from 0.05 to 0.4, with an interval of 0.01 (a total of 36 thresholds were defined). Moreover, the area under the curve (AUC) across 0.05 < S < 0.4 was calculated for each metric to provide a summarized scalar to detect network topological metric changes, which was thought to be more sensitive and reliable than a single threshold.

### 2.5 Network‐based statistical analysis

We used the network‐based‐statistical (NBS) (http://www.nitrc.org/projects/nbs/) approach to further localize altered morphologic connections in the GM network.^27^ First, the nodes with between‐group differences in nodal characteristics were selected to form a sub‐connection matrix. Then, a primary threshold (threshold = 3.2, uncorrected *p* < 0.001) was defined to extract connected components that consisted of a set of suprathreshold connections between pairs of nodes. Finally, we used the permutation test to assess the significance of each component based on its size. More specifically, we repeated the permutation procedure 5000 times and recorded the maximum component size distributions from each permutation. For a connected component of size k, we counted the number of permutations in which the component size was equal to or higher than the identified size of *k*. The corrected *p*‐value was then calculated by dividing this number by the number of permutations (5000).

### 2.6 Statistical analysis

The Shapiro–Wilk (S–W test) was conducted to test the normal distribution of continuous variables. Quantitative variables were tested by two‐sample *t*‐test, and qualitative variables were tested by a chi‐square test. The demographic and clinical data between SMA and HCs were compared using a two‐sample *t*‐test or chi‐square test as appropriate. A two‐tailed *p* < 0.05 was considered to be significant. To compare the AUC of global and nodal network metrics between groups, a non‐parameter permutation test (10,000 permutations) was used.^26^ For each metric, the AUC values were randomly reassigned to two groups, and the mean difference between groups was calculated. And we repeated the randomization process 10,000 times; the 95th percentile of each distribution was used as the threshold for significance testing. To address the multiple comparison correction for nodal metrics, the Benjamini–Hochberg false discovery rate (BHFDR) with a significance value of 0.05 was performed.^28^ Finally, to investigate whether the significant global and nodal network metrics were related to the clinical variables (disease duration and HFMSE scores), Pearson's partial correlation analysis was also conducted in the SMA group with age and gender as covariates (*p* < 0.05).

### 2.7 Reproducibility analysis

To validate the reproducibility of our findings, we adopted two measurements as follows: the variant of the KLDs, Jesen–Shannon divergence‐based similarity (JSDs),^29^, ^30^ was used to construct an individual similarity network in our study to evaluate the effect of different choices of similarity measures; and an additional brain parcellation atlas, the Hammersmith atlas with 83 regions,^31^, ^32^ was adopted to assess the effect of different parcellation schemes.

## 3 RESULTS

### 3.1 Demographic and clinical characteristics

In our study, 38 SMAs and 38 HCs were included. The age and gender were matched between SMA and HCs. Demographic and clinical information is shown in Table 1.

**TABLE 1:** Demographic and clinical features of SMA and HCs.

	SMA^38^ (Mean ± SD)	HCs^38^ (Mean ± SD)	Statistics	*p* value
Age	10.37 ± 3.81 (5–17)	10.30 ± 3.54 (6–20)	−0.078	0.938
Sex (M/F)	24:14	24:14	0	1
SMA type (2/3)	20:18	NA	NA	NA
Age of onset	2.55 ± 2.59 (0.5–12)	NA	NA	NA
Disease duration	7.78 ± 3.61 (2–16.5)	NA	NA	NA
HFMSE score	27.53 ± 22.69 (0–65)	NA	NA	NA

Abbreviations: HCs, healthy controls; HFMSE, Hammersmith Functional Motor Scale Expanded; SMA, spinal muscular atrophy.

### 3.2 Global and nodal network properties in SMA

**FIGURE 1:**
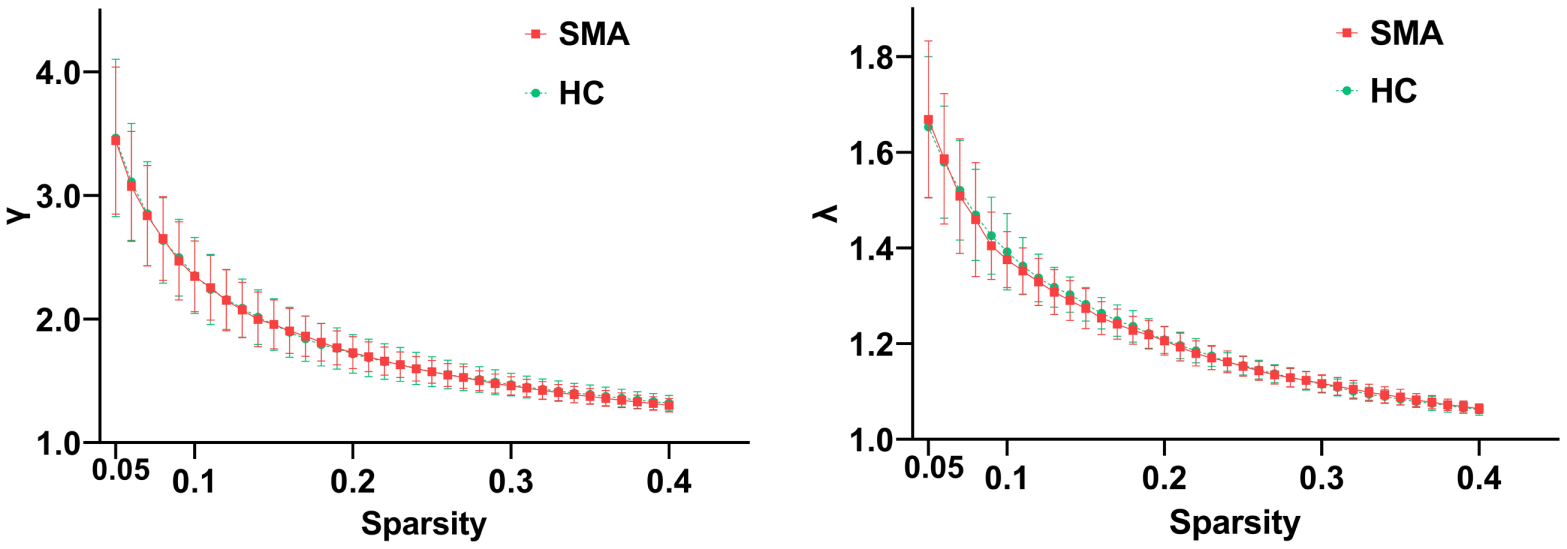
Small‐world topological properties (*γ* > 1 and *λ* ≈ 1) of the gray matter network in SMA and HCs. HCs, healthy controls; SMA, spinal muscular atrophy; *γ*, normalized clustering coefficient; *λ*, normalized characteristic path length.

In the defined threshold range, both groups showed small‐world topology in the KLDs‐based morphological brain network with *γ* > 1 and *λ* ≈ 1 (Figure 1). However, no significant differences between SMA and HCs were found in the AUC of global network properties, including *C*_p_ (*p* = 0.094), *L*_p_ (*p* = 0.459), γ (*p* = 0.436), *λ* (*p* = 0.294), *σ* (*p* = 0.494), Eg (*p* = 0.242), and Eloc (*p* = 0.078), and the details are summarized in Table S1 and Figure S1.

**FIGURE 2:**
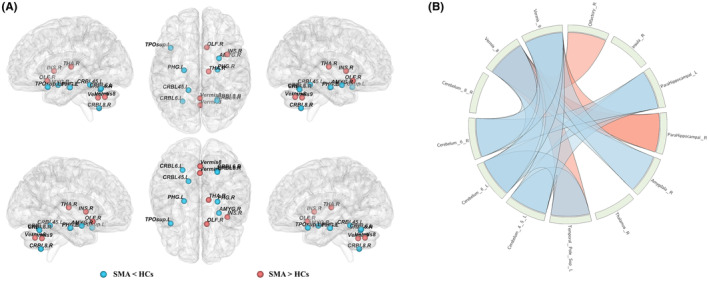
The brain regions showing significant differences in nodal topological properties and network connectivity between SMA and HCs. (A) SMA‐related alterations in nodal topological properties. Red (blue) nodes represent brain regions with increased (decreased) nodal topological properties in SMA. Correction for multiple comparisons was performed using the Benjamini–Hochberg false discovery rate (*p* < 0.05). (B) SMA‐related alterations in the network connectivity. The hypoconnectivity network consists of 8 nodes and 12 edges, and the hyperconnectivity network consists of 5 nodes and 4 edges. Red (blue) connecting lines represent hyperconnectivity (hypoconnectivity) in SMA. AMYG.R, right amygdala; CRBL45.L, left cerebellum IV‐V; CRBL6.L, left cerebellum VI; CRBL6.R, right cerebellum VI; CRBL8.R, right cerebellum VII; HCs, healthy controls; INS.R, right insula; OLF.R, right olfactory cortex; PHG.L, left parahippocampal gyrus; PHG.R, right parahippocampal gyrus; SMA, spinal muscular atrophy; THA.R, right thalamus; TPOsup.L, superior temporal gyrus; Vermis 8, cerebellar vermis VII; Vermis 9, cerebellar vermis IX.

We identified some regions showing significant differences in nodal network properties (*p* < 0.05, FDR‐corrected) and summarized the detailed information in Table 2 and Figure S2. Compared with HCs, individuals with SMA showed increased nodal degree and efficiency in the right insula, right thalamus, and cerebellar vermis VII and IX, and decreased nodal degree and efficiency in the bilateral parahippocampal gyrus, right amygdala, left superior temporal gyrus, left cerebellar lobule IV–V, and bilateral cerebellar lobule VI. In addition, individuals with SMA also showed increased nodal degree in the right olfactory cortex and decreased nodal degree in the right cerebellar lobule VII (Figure 2A). No significant differences in nodal betweenness were found between groups.

**TABLE 2:** The mean, standard deviation, and *p* value of the brain regions with significant difference between SMA and HCs in nodal topological properties.

Brain region	Nodal Degree			Nodal Efficiency			Nodal Betweenness		
	SMA (Mean ± SD)	HC (Mean ± SD)	*p* value	SMA (Mean ± SD)	HC (Mean ± SD)	*p* value	SMA (Mean ± SD)	HC (Mean ± SD)	*p* value
**SMA > HC**									
Olfactory_R	10.1836 ± 2.3434	8.6763 ± 2.2833	**0.0313**	0.1801 ± 0.0162	0.1707 ± 0.0186	0.0702	75.5958 ± 42.8791	69.8276 ± 29.0747	0.4353
Insula_R	5.2788 ± 2.8793	3.1847 ± 1.6223	**0.0058**	0.1393 ± 0.0269	0.1157 ± 0.0266	**0.0039**	15.2284 ± 22.2428	7.0303 ± 9.7669	0.1846
Thalamus_R	6.7003 ± 3.1856	4.7441 ± 3.0538	**0.0419**	0.1507 ± 0.0284	0.1283 ± 0.0363	**0.0335**	33.2634 ± 20.0743	26.2625 ± 23.3701	0.3457
Vermis_8	4.4034 ± 1.4613	3.2479 ± 1.5435	**0.0058**	0.1334 ± 0.0194	0.1099 ± 0.0232	**0.0039**	26.1046 ± 19.1682	20.7220 ± 17.7418	0.3489
Vermis_9	8.0841 ± 2.6125	5.1679 ± 2.3504	**0.0058**	0.1632 ± 0.0240	0.1341 ± 0.0268	**0.0039**	36.5399 ± 29.8446	28.9520 ± 26.0951	0.3489
**SMA < HC**									
ParaHippocampal_L	11.6084 ± 4.3600	13.7224 ± 2.1043	**0.0367**	0.1832 ± 0.0463	0.2030 ± 0.0127	**0.0383**	25.7506 ± 20.5436	26.5983 ± 12.9563	0.4888
ParaHippocampal_R	9.2943 ± 2.3557	10.8251 ± 1.6875	**0.0155**	0.1758 ± 0.0186	0.1865 ± 0.0113	**0.0188**	18.0431 ± 11.2859	21.0865 ± 18.3388	0.3843
Amygdala_R	1.1463 ± 1.0353	2.0847 ± 1.4620	**0.0232**	0.0606 ± 0.0345	0.0822 ± 0.0367	**0.0485**	10.2818 ± 13.3620	9.2039 ± 10.8605	0.4775
Temporal_Pole_Sup_L	11.3567 ± 3.0130	13.4186 ± 1.7697	**0.0093**	0.1890 ± 0.0208	0.2019 ± 0.0108	**0.0039**	21.6397 ± 12.4801	29.7928 ± 16.8888	0.1846
Cerebelum_4_5_L	3.5733 ± 1.8980	5.5322 ± 2.5656	**0.0058**	0.1177 ± 0.0288	0.1430 ± 0.0242	**0.0039**	10.1071 ± 12.5907	17.9171 ± 17.3710	0.1846
Cerebelum_6_L	0.8133 ± 0.6580	1.2972 ± 0.7297	**0.0284**	0.0497 ± 0.0317	0.0758 ± 0.0238	**0.0039**	8.4107 ± 9.8685	9.3551 ± 11.4436	0.4775
Cerebelum_6_R	0.5847 ± 0.4097	0.9597 ± 0.7441	**0.0327**	0.0380 ± 0.0272	0.0602 ± 0.0311	**0.0099**	5.1499 ± 8.0021	3.3700 ± 4.7827	0.3497
Cerebelum_8_R	6.6695 ± 2.8437	8.8897 ± 3.8553	**0.0284**	0.1559 ± 0.0233	0.1715 ± 0.0297	0.0503	13.1628 ± 19.4513	19.1064 ± 14.5122	0.3457

*Note*: *p* values shown in bold font were considered to be significant (*p* < 0.05, FDR‐corrected).

Abbreviation: HCs, healthy controls; SD, standard deviation; SMA, spinal muscular atrophy.

Further, comparisons between SMA subgroups were also performed. Our findings showed that both subgroups exhibited small‐world properties, but no significant differences were found between SMA type 2 and type 3 for both global and nodal topological properties (see Appendix S1).

### 3.3 SMA‐related alterations in morphological connectivity

We used the NBS method to identify two connected sub‐networks that were significantly altered in SMA. As shown in Figure 2B, the hypoconnectivity network consisted of 8 nodes and 12 edges (corrected *p* < 0.001), and the hyperconnectivity network consisted of 5 nodes and 4 edges (corrected *p* = 0.001). Further, the altered morphological network connectivity was mainly located in the cortical–limbic‐cerebellum circuit (Table 3).

**TABLE 3:** The mean, standard deviation, and *p* value of the altered morphological connectivity between SMA and HCs.

	SMA (Mean ± SD)	HC (Mean ± SD)	*p* value
**SMA > HC**			
Olfactory_R – Vermis_8	0.8953 ± 0.0707	0.8078 ± 0.1071	0.001
Parahippocampal_R – Vermis_8	0.7997 ± 0.1680	0.5787 ± 0.1872	0.001
Temporal_Pole_Sup_L – Vermis_8	0.6504 ± 0.1570	0.4915 ± 0.1330	0.001
Parahippocampal_R – Vermis_9	0.8276 ± 0.1443	0.6781 ± 0.1818	0.001
**SMA < HC**			
Parahippocampal_L – Cerebellum_4_5_L	0.5080 ± 0.1999	0.6874 ± 0.1130	<0.001
Temporal_Pole_Sup_L – Cerebellum_4_5_L	0.5306 ± 0.1564	0.6676 ± 0.1225	<0.001
Parahippocampal_L – Cerebellum_6_L	0.3018 ± 0.1764	0.4562 ± 0.1572	<0.001
Temporal_Pole_Sup_L – Cerebellum_6_L	0.8244 ± 0.0516	0.8331 ± 0.0487	<0.001
Parahippocampal_L – Cerebellum_6_R	0.2348 ± 0.1589	0.3662 ± 0.1761	<0.001
Amygdala_R – Vermis_8	0.6226 ± 0.1813	0.8206 ± 0.1191	<0.001
Cerebellum_6_L – Vermis_8	0.6195 ± 0.1847	0.8123 ± 0.1083	<0.001
Cerebellum_6_R – Vermis_8	0.5407 ± 0.2192	0.8106 ± 0.0906	<0.001
Amygdala_R – Vermis_9	0.5821 ± 0.1941	0.7604 ± 0.1539	<0.001
Cerebellum_4_5_L – Vermis_9	0.7431 ± 0.1487	0.8359 ± 0.0829	<0.001
Cerebellum_6_L – Vermis_9	0.5690 ± 0.1924	0.7553 ± 0.1042	<0.001
Cerebellum_6_R – Vermis_9	0.4818 ± 0.2044	0.7081 ± 0.1136	<0.001

Abbreviations: HCs, healthy controls; SD, standard deviation; SMA, spinal muscular atrophy.

### 3.4 Correlation analysis between network properties and clinical characteristics

**FIGURE 3:**
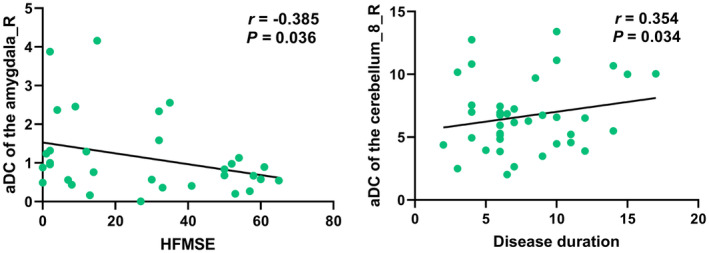
Correlation analysis results between the altered nodal metrics and clinical characteristics in SMA. aDC, the AUC values of degree centrality; HFMSE, Hammersmith Functional Motor Scale Expanded; SMA, spinal muscular atrophy.

The detailed results of the partial correlation analysis are shown in Figure 3. The nodal degree of the right cerebellar lobule VII was positively correlated with the disease duration (*r* = 0.354, *p* = 0.034, uncorrected), and the nodal degree of the right amygdala was negatively correlated with the HFMSE scores (*r* = −0.385, *p* = 0.036, uncorrected).

### 3.5 Reproducibility of findings

The main results of JSDs‐based morphological network analysis were similar to those of the KLDs method (see Appendix S1). The individual‐level morphological network also exhibited small‐world organization in both groups (Figure S3). Moreover, the brain regions with significant differences in nodal network properties were consistently identified in SMA, mainly involving the right olfactory cortex, right insula, bilateral parahippocampal gyrus, right amygdala, right thalamus, left superior temporal gyrus, left cerebellar lobule IV–V, bilateral cerebellar lobule VI, right cerebellar lobule VII, and cerebellar vermis VII and IX (Table S2; Figure S4).

Similarly, the small‐world properties of the brain morphological network were observed in both groups when using the Hammersmith‐83 atlas for brain parcellation (Figure S5). Further analyses based on the Hammersmith‐83 atlas showed that no significant differences were found in the AUC of global network properties between groups, but significantly altered nodal network properties of brain regions such as the precentral gyrus, orbital gyrus, anterior and posterior cingulate gyrus, temporal gyrus, parahippocampal gyrus, nucleus accumbens, thalamus, caudate nucleus, and cerebellum were found in SMA (Figure S6; Tables S3 and S4).

## 4 DISCUSSION

To the best of our knowledge, our study is the first to construct an individual‐level whole‐brain morphological brain network using both KLDs and JSDs methods in SMA, and three main findings were obtained: (1) the small‐world topological property of the brain morphological network consistently observed in SMA was independent of the choices of similarity measures and brain parcellation schemes; (2) significantly altered nodal properties rather than global topological properties were found in SMA, and the involved brain regions were mainly located in the cortical–limbic‐cerebellum circuitry; (3) the nodal properties of the right cerebellar lobule VII and right amygdala were associated with the clinical characteristics (disease duration and motor function severity) in SMA.

The human brain is generally thought to be an economic network that supports specialized information processing in local regions and distributed information processing across the whole brain. The economic model of brain network organization emphasized the topological properties, including small‐world, modularity, and rich hubs in both functional and structural networks, by using the graph‐theory approach.^33^, ^34^ In line with these views, the small‐world properties of GM networks consistently revealed by both KLD‐based and JSD‐based networks in our study further highlight the optimal balance between segregation and integration in morphological networks. However, no significant differences in all global network properties were observed between SMA and HCs, and we proposed several possible interpretations for this phenomenon. First, the average age of the individuals with SMA in our study was about 10 years, and the greater the plasticity of adolescent brain development, the more likely it is to prioritize the maintenance of global topological organization over nodal topological properties.^35^ Second, previous studies have found the coexistence of an increase and a reduction of GM volume in SMA.^9^, ^10^ The classical explanation for this finding was that the decreased volume was the direct result of neurodegeneration, while the increased volume may reflect the compensatory response.^21^ Thus, the effects of the neurodegenerative process and concomitant disease‐adaptive compensatory mechanism may overlap and contribute to the similar global network topology observed in HCs. Overall, our findings of comparable global network topology between groups may need to take into account the ongoing brain development of adolescent patients and the addictive effect of disease on it.

In contrast to the preserved overall network topology, significantly altered nodal centrality and efficiency of the cerebellum were found in SMA. This finding of the involvement of the cerebellum was supported by emerging evidence from animal models, autoptic, and brain MRI studies in SMA. A recent mouse model of SMA study using a muti‐technique approach^36^ presented a comprehensive picture of cerebellar pathology in SMA, including loss of cerebellar volume, degeneration of Purkinje cells, dysfunction in cerebellar afferent tracts, and alterations in intrinsic properties of cerebellar output neurons. Meanwhile, limited post‐mortem studies and clinical brain MRI studies^6^, ^19^ revealed neurodegeneration, neuron ballooning, and neuronophagia, as well as progressive atrophy in the cerebellum of severe SMA. Moreover, a recent neuroimaging study of cerebellar structural analysis^9^ also reported decreased volumes of cerebellar lobules VIIIB, IX, and X in SMA. Thus, these changes suggest that cerebellum involvement may be a common finding in SMA. In fact, the cerebellum has long been thought to be critical for intact motor function based on extensive connectivity with cortical and subcortical regions, and the cerebellum pathology also contributes to the clinical manifestations of another similar motor neuron disease, amyotrophic lateral sclerosis (ALS). In particular, the cerebellum was considered to play a compensatory role for primary motor region degeneration in the ALS‐related pathological process.^37^, ^38^ Unlike this, the SMN protein, which plays a crucial role in SMA‐related pathology, was found to be highly expressed in Purkinje cells and deep cerebellar nuclei in both humans and rats.^2^, ^39^, ^40^, ^41^ Thus, the cerebellum pathology in SMA is more likely to be the direct result of the reduced SMN expression. On the other hand, these differences may also emphasize the disease‐specific cerebellar pathology among motor neuron diseases.

Interestingly, our study also showed that regions with nodal property alterations involved some non‐motor structures, including the parahippocampal gyrus, amygdala, and insula. These brain regions are crucial in the limbic system, which is essential to emotional and cognitive regulation. It seemed difficult for us to understand the involvement of nonmotor‐related limbic structures. However, more attention has been paid to the cognitive profiles in SMA. Contrary to frequently observed cognitive deficits in other neuromuscular diseases, such as ALS,^42^ Duchenne muscular dystrophy^43^ and myotonic dystrophy,^44^ it seemed that overall cognitive performance was comparable or even superior to controls in SMA types 2 and 3. Most studies have attributed this finding to the compensational mechanism hypothesis, which proposes that motor impairment could promote enhanced cognitive adaptation.^45^, ^46^ Thus, combined with our study, we speculated that the topological properties and alteration patterns of limbic structures serve as key neural network mechanisms to maintain normal or above‐average cognitive function. In the future, a comprehensive and specific cognitive evaluation will help to better understand the mechanism. In addition, the association between amygdala and HFMSE scores suggested that the amygdala may have an effect on movement in SMA. Indeed, as the key hub of the limbic system, the amygdala plays an important role in the generation, recognition, and regulation of emotion. Previous studies have shown that individuals with SMA also suffer from emotional behaviors such as fatigue and depressive feelings.^47^, ^48^ Thus, a possible explanation for this association between the amygdala and motor disability is the indirect effect of emotion.

Aside from the above findings, compared with HCs, individuals with SMA also showed extensive alterations in other brain regions, including the temporal gyrus, olfactory gyrus, and thalamus. As a deep cerebral structure, the thalamus acts as a relay center between cortical and subcortical regions to subserve both motor and sensory functions. Neuropathological studies in SMA also reported the prominent involvement of thalamic and sensory neurons.^3^, ^6^ Thus, consistent with previous studies, our findings lend support to the view that the thalamus is involved in SMA.

Finally, the definition of nodes and edges was supposed to be important in a graph‐based brain network. In our study, we employed two brain atlases (AAL‐116 and Hammersmith‐83) to parcellate the brain region and two similarity measurements (KLDs and JSDs) to estimate interregional morphological similarity. Indeed, the findings of the preserved global network topology consistently observed in SMA are independent of the choices of similarity measures and brain parcellation schemes. Meanwhile, the brain regions with significant changes in nodal network properties based on AAL‐116 were partially similar to those based on the Hammersmith‐83 atlas, such as the temporal gyrus, parahippocampal gyrus, thalamus, and cerebellum, which not only increase the stability and reproducibility of these findings but also lend support to the critical role of the cortical–limbic‐cerebellar circuit in SMA.

### 4.1 Limitations

Some limitations of this study need to be noted. First, although the results of KLDs‐based and JSDS‐based methods were shown to be similar and reliable, we used only GM volume to construct individual morphological brain networks. Other morphological features (such as cortical thickness from surface‐based analysis) and different imaging modalities (such as diffusion tensor imaging and functional MRI) could be combined to help us fully understand the neural network mechanisms underlying SMA. Second, it was worth noting that the results of correlation analysis did not survive FDR correction; thus, future studies with a larger sample size may be more able to increase the statistical power. Third, whether there are different pathological mechanisms between different clinical phenotypes remains unclear. According to our findings, no significant differences were found between SMA type 2 and type 3. However, this result could not exclude the results bias caused by age differences determined primarily by different phenotypes and the small sample size. Thus, direct comparisons between SMA subtypes with a larger sample size and age‐matched HCs were needed to further reveal the unique pathological mechanisms underlying different clinical phenotypes. Finally, since we only evaluated the motor function of individuals with SMA, a more detailed study of clinical characteristics, especially cognitive function and mood state, will be helpful to elucidate the mechanisms of the disease.

## 5 CONCLUSION

In conclusion, our study of topological GM network analysis revealed preserved global properties but extensively altered nodal properties in SMA. Among these brain regions, the cerebellar and limbic networks may function as key pathogenesis hubs underlying SMA. These findings contribute to the further understanding of the large‐scale neural network mechanism and provide a possible brain target for the treatment of the disease in the future.

## AUTHOR CONTRIBUTIONS

LYF and NHR: conception, study design, data acquisition, statistical analysis, writing‐original draft, writing‐review and editing. XP, SWQ, YMZ, YC, SS, and QL: acquisition, analysis, and interpretation of data. LYJ, TW, and YZY: conception, study design, writing‐review and editing. LYJ and CYQ: conception, study design, funding acquisition, formal analysis, writing‐review and editing. All authors read and approved the final manuscript.

## FUNDING INFORMATION

This research was supported by the Natural Science Fund Project of Guangdong Province (grant number 2022A1515011910) and the National Natural Science Foundation of China (grant number 82001439).

## CONFLICT OF INTEREST STATEMENT

The authors have no potential conflicts of interest to report.

## CONSENT FROM THE PARTICIPANT

Written informed consent was obtained from all subjects.

## Supporting information


Appendix S1.

## Data Availability

The data that support the findings of this study are available from the corresponding author on reasonable request.
