# Supplementary material for: Disrupted individual‐level morphological brain network in spinal muscular atrophy types 2 and 3
Source: CNS Neurosci Ther. 2024 Jun 17;30(6):e14804. doi: 10.1111/cns.14804 (PMC11183166; doi:10.1111/cns.14804)

***Supplementary materials***

**Figure S1. Comparisons of global topological properties between SMA and HCs based on KLDs method.**

**
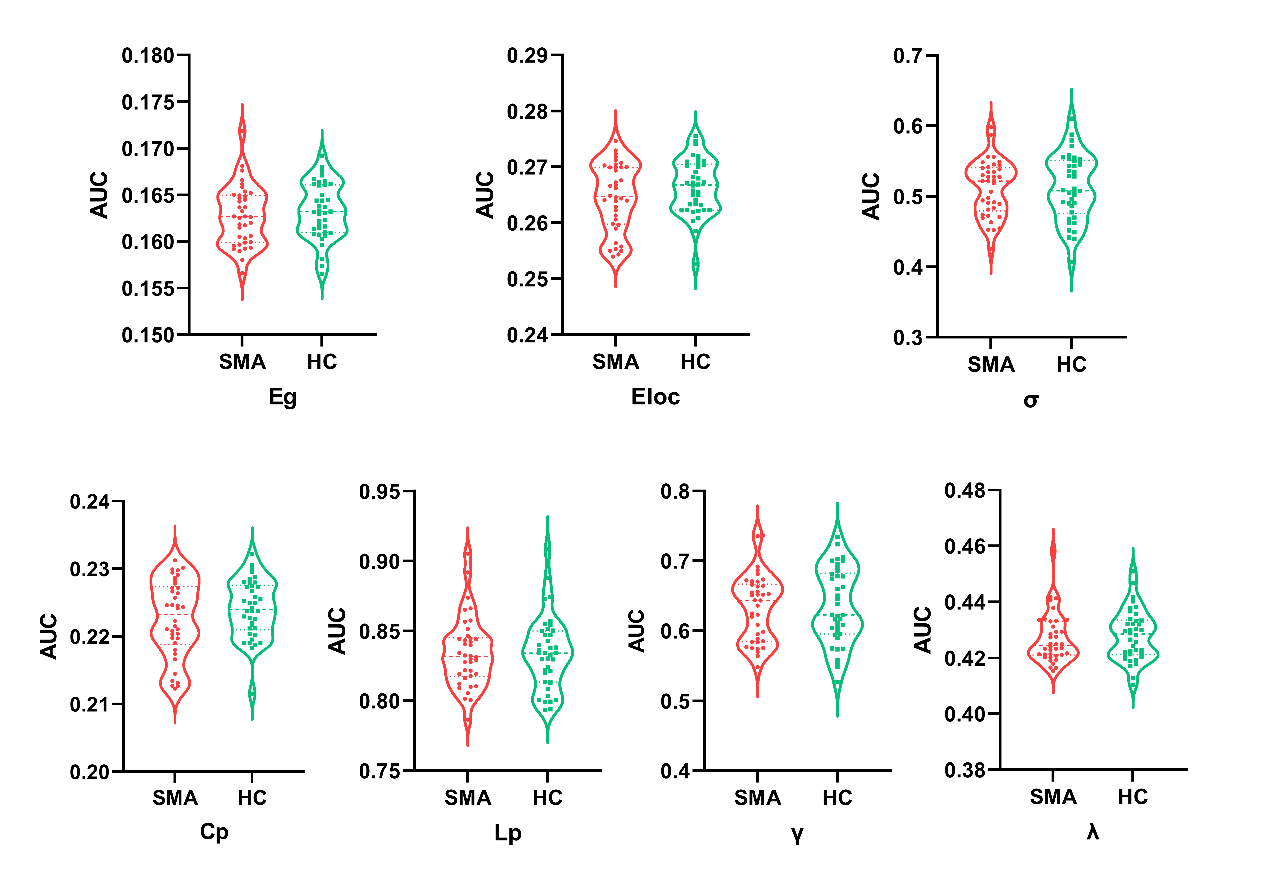
**

**Figure S2. Between-group differences in the AUC of nodal degree in SMA and HCs based on KLDs method.**

**
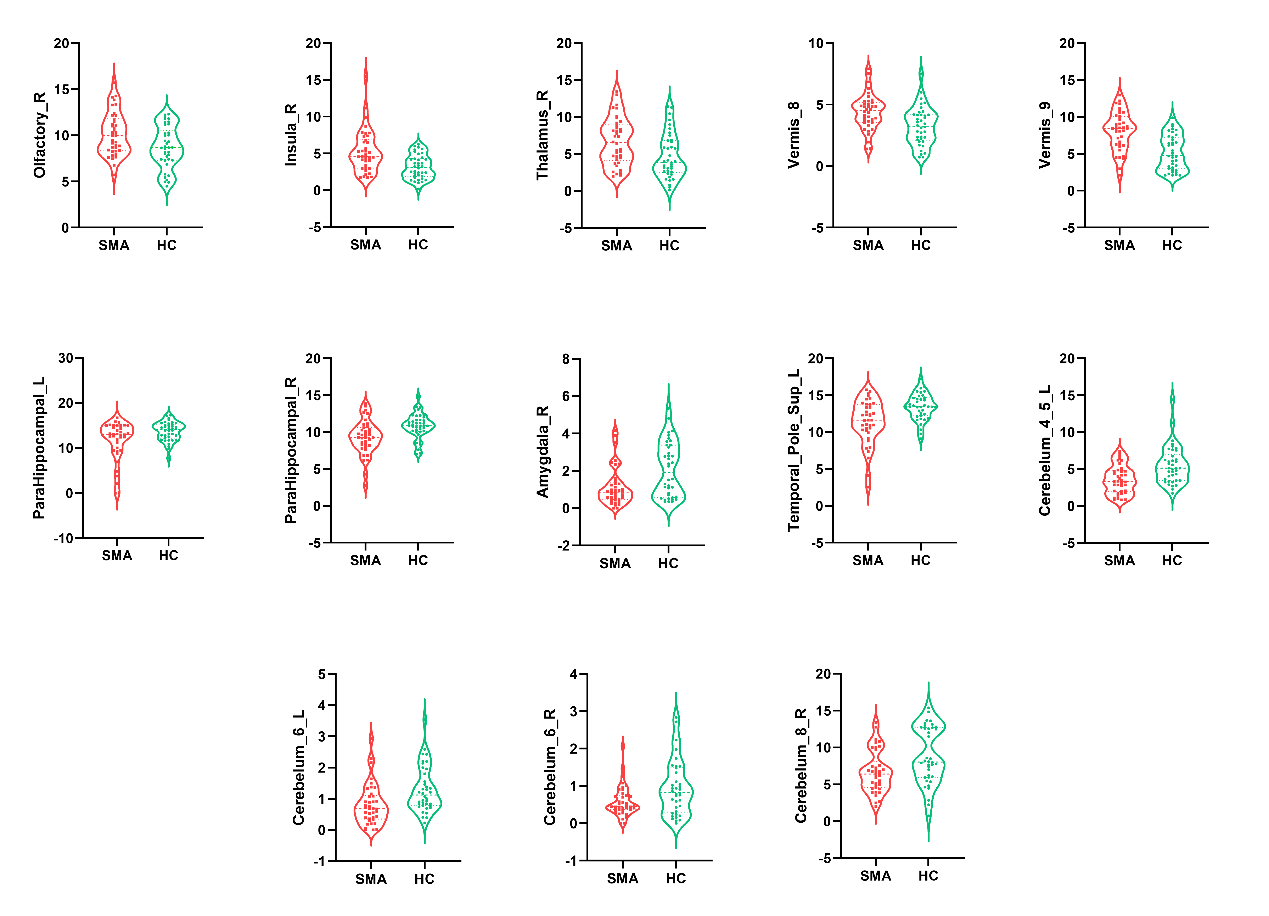
**

**Figure S3. Small-world topological properties (γ＞1 and λ≈1) of the gray matter network in SMA and HCs based on JSDs method.**


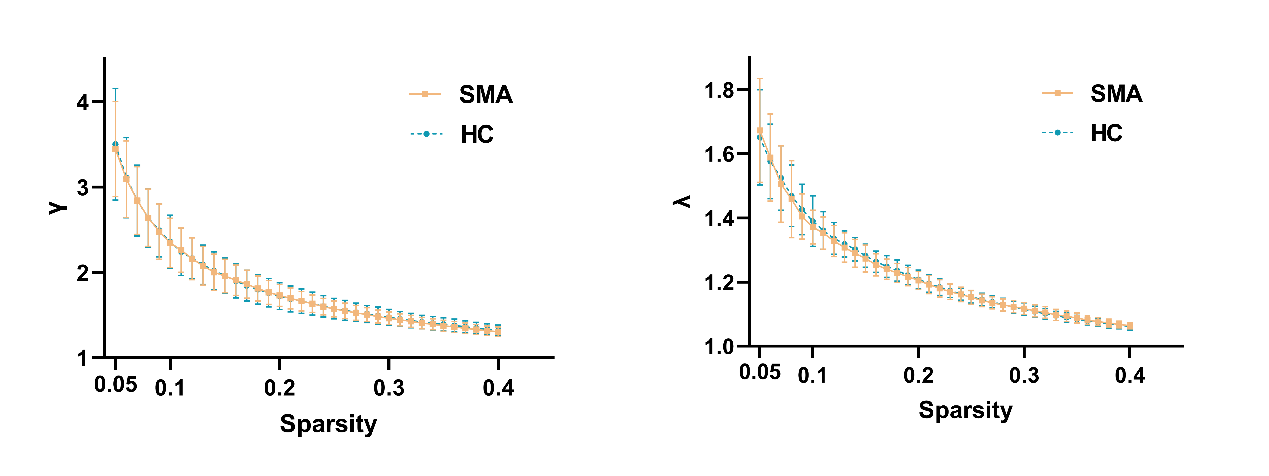


**Figure S4. The brain regions showing significant differences in nodal topological properties between SMA and HCs based on JSDs method.**


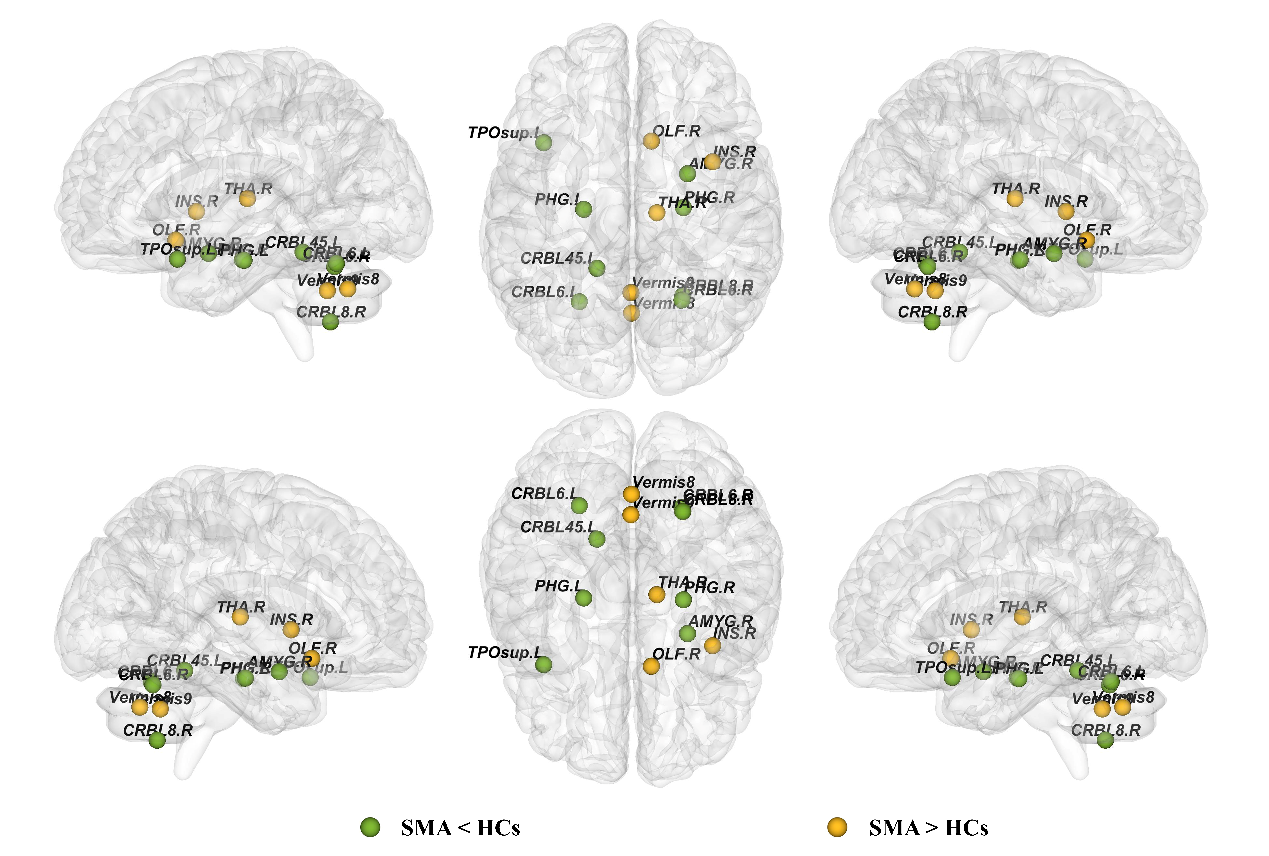


**Figure S5. Small-world topological properties (γ＞1 and λ≈1) of the gray matter network in SMA and HCs based on KLDs method by using Hammersmith atlas.**

**
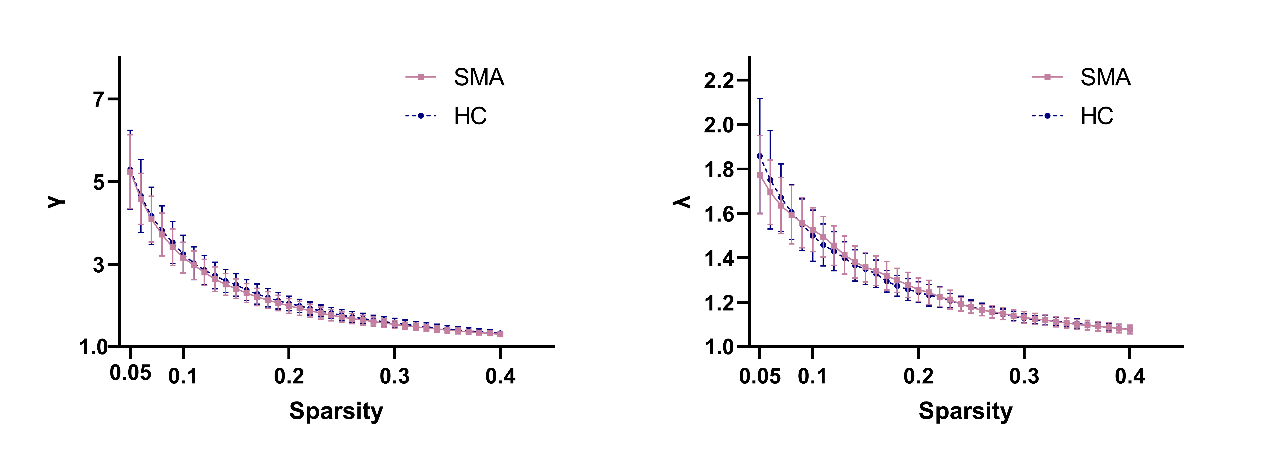
**

**Figure S6. Comparisons of global topological properties between SMA and HCs based on KLDs method by using Hammersmith atlas.**

**
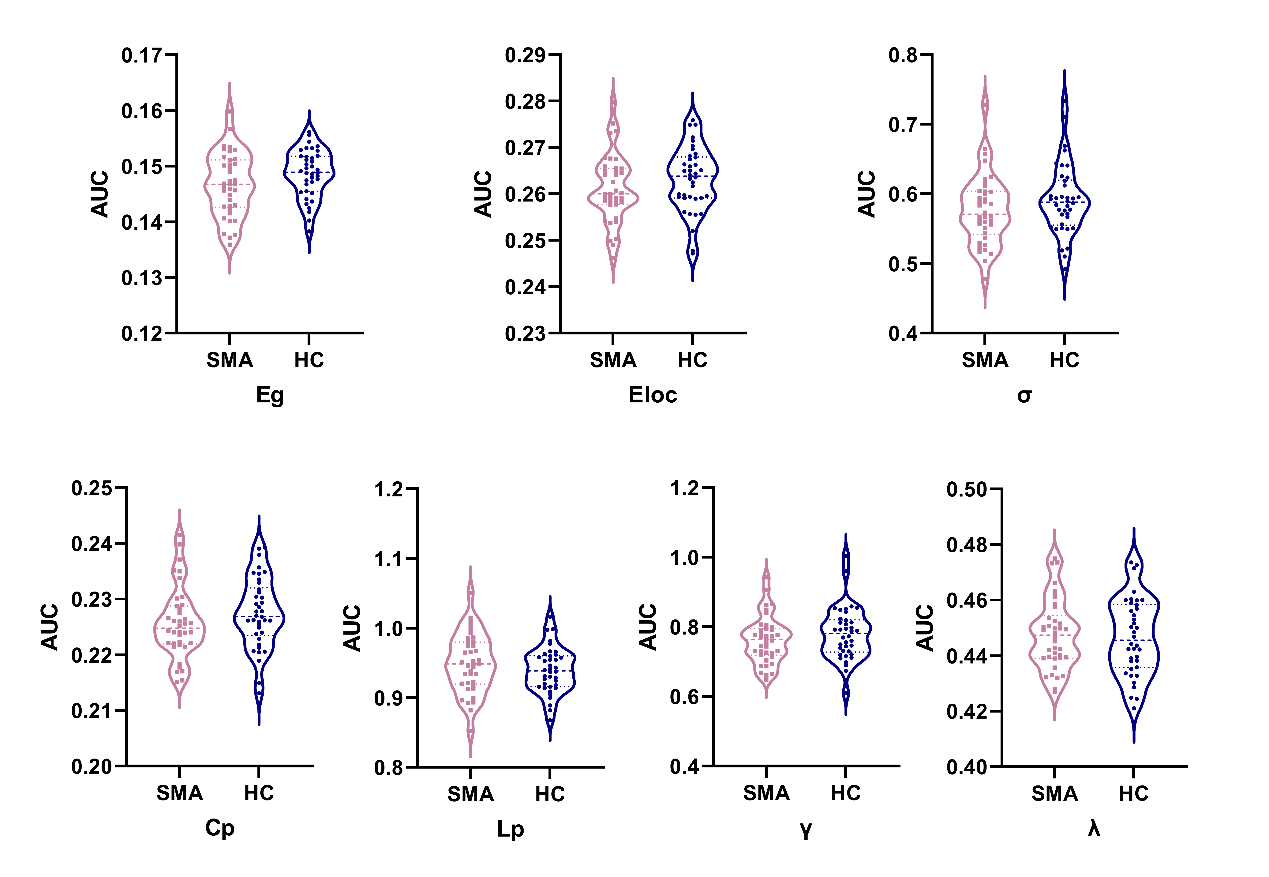
**

**Table S1. Between-group differences in the global topological properties** **in SMA and HCs based on KLDs method**.

|  | SMA (Mean ± SD) | HC (Mean ± SD) | *P* value |
| --- | --- | --- | --- |
| aCp | 0.2225 ± 0.0057 | 0.2241 ± 0.0043 | 0.0940 |
| aLp | 0.8344 ± 0.0249 | 0.8339 ± 0.0261 | 0.4593 |
| aGamma | 0.6329 ± 0.0474 | 0.6349 ± 0.0540 | 0.4362 |
| aLambda | 0.4270 ± 0.0088 | 0.4281 ± 0.0091 | 0.2941 |
| aSigma | 0.5115 ± 0.0393 | 0.5119 ± 0.0464 | 0.4942 |
| aEg | 0.1628 ± 0.0032 | 0.1633 ± 0.0030 | 0.2418 |
| aEloc | 0.2646 ± 0.0060 | 0.2664 ± 0.0049 | 0.0776 |

**Table S2. The mean, standard deviation and *P* value of the brain regions with significant differences between SMA and HCs in nodal topological properties based on JSDs method.**

|  | Nodal Degree | | |  | Nodal Efficiency | | |  | Nodal Betweenness | | |
| --- | --- | --- | --- | --- | --- | --- | --- | --- | --- | --- | --- |
| brain region | SMA  (Mean ± SD) | HC  (Mean ± SD) | *P* value |  | SMA  (Mean ± SD) | HC  (Mean ± SD) | *P* value |  | SMA  (Mean ± SD) | HC  (Mean ± SD) | *P* value |
| SMA > HC | | | | | | | | | | | |
| Olfactory_R | 10.1932 ± 2.3393 | 8.6959 ± 2.2914 | **0.0348** |  | 0.1802 ± 0.0162 | 0.1708 ± 0.0185 | 0.0718 |  | 74.3316 ± 43.3579 | 67.9580 ± 29.2034 | 0.4053 |
| Insula_R | 5.3087 ± 2.8681 | 3.2261 ± 1.6283 | **0.0077** |  | 0.1394 ± 0.0267 | 0.1159 ± 0.0265 | **0.0039** |  | 15.5477 ± 22.8940 | 7.3952 ± 10.0521 | 0.2088 |
| Thalamus_R | 6.6867 ± 3.1693 | 4.7344 ± 3.0498 | **0.0384** |  | 0.1506 ± 0.0283 | 0.1281 ± 0.0363 | **0.0219** |  | 32.8936 ± 19.5690 | 26.2199 ± 23.5100 | 0.3458 |
| Vermis_8 | 4.4923 ± 1.4790 | 3.3018 ± 1.5627 | **0.0162** |  | 0.1341 ± 0.0195 | 0.1105 ± 0.0233 | **0.0039** |  | 25.2794 ± 19.0270 | 20.1199 ± 17.1351 | 0.3517 |
| Vermis_9 | 8.0928 ± 2.6095 | 5.1921 ± 2.3606 | **0.0058** |  | 0.1631 ± 0.0241 | 0.1342 ± 0.0270 | **0.0039** |  | 35.3754 ± 28.5119 | 28.6114 ± 25.8191 | 0.3838 |
| SMA < HC | | | | | | | | | | | |
| ParaHippocampal_L | 11.6272 ± 4.3346 | 13.7389 ± 2.0607 | **0.0348** |  | 0.1834 ± 0.0462 | 0.2031 ± 0.0124 | **0.0336** |  | 25.5080 ± 20.0998 | 26.6375 ± 12.8579 | 0.4928 |
| ParaHippocampal_R | 9.3513 ± 2.3456 | 10.9038 ± 1.6587 | **0.0166** |  | 0.1762 ± 0.0185 | 0.1869 ± 0.0112 | **0.0174** |  | 17.8768 ± 11.1438 | 20.8403 ± 17.9599 | 0.4001 |
| Amygdala_R | 1.1539 ± 1.0450 | 2.1143 ± 1.4950 | **0.0166** |  | 0.0604 ± 0.0343 | 0.0826 ± 0.0368 | 0.0506 |  | 10.2398 ± 13.4788 | 9.3007 ± 11.0613 | 0.4928 |
| Temporal_Pole_Sup_L | 11.3009 ± 2.9952 | 13.3731 ± 1.7770 | **0.0145** |  | 0.1888 ± 0.0205 | 0.2017 ± 0.0108 | **0.0039** |  | 21.5230 ± 12.4052 | 29.7494 ± 16.7711 | 0.2068 |
| Cerebelum_4_5_L | 3.6059 ± 1.9026 | 5.5869 ± 2.5602 | **0.0058** |  | 0.1179 ± 0.0288 | 0.1432 ± 0.0243 | **0.0039** |  | 10.7887 ± 13.0060 | 18.4881 ± 17.6048 | 0.2088 |
| Cerebelum_6_L | 0.8230 ± 0.6656 | 1.3181 ± 0.7382 | **0.0290** |  | 0.0502 ± 0.0316 | 0.0763 ± 0.0240 | **0.0039** |  | 8.7457 ± 10.2018 | 9.1896 ± 11.3149 | 0.4978 |
| Cerebelum_6_R | 0.5925 ± 0.4196 | 0.9767 ± 0.7558 | **0.0348** |  | 0.0385 ± 0.0271 | 0.0607 ± 0.0312 | **0.0174** |  | 5.4724 ± 8.2756 | 3.4208 ± 4.8735 | 0.3458 |
| Cerebelum_8_R | 6.6989 ± 2.8506 | 8.9152 ± 3.8623 | **0.0348** |  | 0.1561 ± 0.0232 | 0.1716 ± 0.0298 | 0.0541 |  | 13.3291 ± 19.1313 | 19.3214 ± 14.6924 | 0.3349 |

**Table S3. The regions in the Hammersmith-83 atlas.**

| **Index** | **Region** | **Class** | **Index** | **Region** | **Class** |
| --- | --- | --- | --- | --- | --- |
| 1; 2 | Hippocampus | Temporal Lobe | 28; 29 | Middle frontal gyrus | Frontal Lobe |
| 3; 4 | Amygdala | Temporal Lobe | 50; 51 | Precentral gyrus | Frontal Lobe |
| 5; 6 | Anterior temporal lobe, medial part | Temporal Lobe | 52; 53 | Straight gyrus | Frontal Lobe |
| 7; 8 | anterior temporal lobe, lateral part | Temporal Lobe | 54; 55 | Anterior orbital gyrus | Frontal Lobe |
| 9; 10 | Parahippocampal and ambient gyri | Temporal Lobe | 56; 57 | Inferior frontal gyrus | Frontal Lobe |
| 11; 12 | Superior temporal gyrus, posterior part | Temporal Lobe | 58; 59 | superior frontal gyrus | Frontal Lobe |
| 13; 14 | Superior temporal gyrus, posterior part | Temporal Lobe | 68; 69 | Medial orbital gyrus | Frontal Lobe |
| 15; 16 | Fusiform gyrus | Temporal Lobe | 70; 71 | Lateral orbital gyrus | Frontal Lobe |
| 30; 31 | Posterior temporal lobe | Temporal Lobe | 72; 73 | Posterior orbital gyrus | Frontal Lobe |
| 82; 83 | Superior temporal gyrus, anterior part | Temporal Lobe | 76; 77 | Subgenual anterior cingulate gyrus | Frontal Lobe |
| 17; 18 | Cerebellum | Posterior Fossa | 78; 79 | Subcallosal area | Frontal Lobe |
| 19 | Brainstem | Posterior Fossa | 80; 81 | Pre-subgenual anterior cingulate gyrus | Frontal Lobe |
| 20; 21 | Insula | Insula and cingulate Gyri | 34; 35 | Caudate nucleus | Central Structures |
| 24; 25 | Cingulate gyrus (gyrus cinguli), anterior part | Insula and cingulate Gyri | 36; 37 | Nucleus accumbens | Central Structures |
| 26; 27 | Cingulate gyurs (gyrus cinguli), posterior part | Insula and cingulate Gyri | 38; 39 | Putamen | Central Structures |
| 64; 65 | Lingual gyrus | Occipital Lobe | 40; 41 | Thalamus | Central Structures |
| 66; 67 | Cuneus | Occipital Lobe | 42; 43 | Pallidum | Central Structures |
| 22; 23 | Lateral remainder of occipital lobe | Occipital Lobe | 44 | Corpus callosum | Central Structures |
| 60; 61 | Postcentral gyrus | Parietal Lobe | 74; 75 | Substantia nigra | Central Structures |
| 62; 63 | Superior parietal gyrus | Parietal Lobe | 45; 46 | Lateral ventricle (excluding temporal horn) | Ventricles |
| 32; 33 | Inferiolateral remainder of parietal lobe | Parietal Lobe | 47; 48 | Lateral ventricle, temporal horn | Ventricles |
|  |  |  | 49 | Third ventricle | Ventricles |

**Table S4. The mean, standard deviation and *P* value of the brain regions with significant differences between SMA and HCs in nodal topological properties based on KLDs method by using Hammersmith atlas.**

| brain region | Nodal Degree | | |  | Nodal Efficiency | | |  | Nodal Betweeness | | |
| --- | --- | --- | --- | --- | --- | --- | --- | --- | --- | --- | --- |
|  | SMA  (Mean ± SD) | HC  (Mean ± SD) | *P* value |  | SMA  (Mean ± SD) | HC  (Mean ± SD) | *P* value |  | SMA  (Mean ± SD) | HC  (Mean ± SD) | *P* value |
|  |  |  |  |  |  |  |  |  |  |  |  |
| SMA > HC |  | | | | | | | | | | |
| Lateral remainder of occipital lobe_R | 7.6526 ± 1.7060 | 6.7608 ± 1.8695 | **0.0326** |  | 0.1689 ± 0.0177 | 0.1601 ± 0.0203 | **0.0494** |  | 21.0220 ± 16.2219 | 15.5825 ± 18.9621 | 0.1891 |
| Nucleus accumbens_L | 1.3878 ± 0.8841 | 1.0195 ± 0.9335 | 0.0861 |  | 0.0644 ± 0.2892 | 0.0501 ± 0.3219 | **0.0487** |  | 7.9703 ± 6.88475 | 5.3530 ± 8.5190 | 0.1481 |
| Thalamus_R | 7.9787 ± 1.8210 | 6.2442 ± 2.1696 | **0.0003** |  | 0.1750 ± 0.0148 | 0.1586 ± 0.0229 | **0.0004** |  | 25.1717 ± 13.9604 | 27.8684 ± 22.6560 | 0.5399 |
| Anterior orbital gyrus_R | 11.0050 ± 0.7419 | 10.2879 ± 0.9451 | **0.0004** |  | 0.2019 ± 0.0060 | 0.1979 ± 0.0070 | **0.0099** |  | 32.7933 ± 15.3083 | 25.6721 ± 13.0386 | **0.0331** |
| SMA < HC | | | | | | | | | | | |
| Anterior temporal lobe, medial part_R | 9.3654 ± 1.8610 | 9.2858 ± 1.4833 | 0.8248 |  | 0.1878 ± 0.0178 | 0.1884 ± 0.0126 | 0.8695 |  | 21.6934 ± 14.6485 | 29.5537 ± 18.5185 | **0.0443** |
| Parahippocampal and ambient gyri_L | 8.0322 ± 2.6073 | 8.778 ± 1.0615 | 0.1117 |  | 0.1738 ± 0.0334 | 0.1849 ± 0.0080 | **0.0499** |  | 11.3094 ± 8.1498 | 12.932 ± 9.2347 | 0.4211 |
| Middle and inferior temporal gyrus_L | 9.2028 ± 1.3861 | 9.7555 ± 1.6851 | 0.1235 |  | 0.1867 ± 0.0134 | 0.1921 ± 0.0174 | 0.1336 |  | 28.3839 ± 19.6861 | 42.0288 ± 23.0614 | **0.0077** |
| Cerebellum_L | 5.1418 ± 1.4332 | 5.7524 ± 1.1976 | **0.0486** |  | 0.1376 ± 0.0221 | 0.1459 ± 0.0162 | 0.0677 |  | 2.4093 ± 2.2514 | 2.5386 ± 1.8543 | 0.7786 |
| Cingulate gyurs, posterior part_L | 7.8386 ± 1.8430 | 8.5866 ± 2.1781 | 0.1051 |  | 0.1754 ± 0.0184 | 0.1830 ± 0.0199 | 0.0789 |  | 11.663 ± 6.87.3 | 16.1365 ± 9.1505 | **0.0183** |
| Caudate nucleus_R | 3.0284 ± 0.6974 | 3.0716 ± 0.6979 | 0.7883 |  | 0.1080 ± 0.0189 | 0.1107 ± 0.0156 | 0.4950 |  | 18.6197 ± 12.7093 | 28.4272 ± 26.0944 | **0.0273** |
| Precentral gyrus_L | 4.5239 ± 1.1304 | 4.9963 ± 0.7455 | **0.0320** |  | 0.1357 ± 0.0166 | 0.1424 ± 0.0102 | **0.0373** |  | 5.2292 ± 5.9218 | 8.0588 ± 7.5558 | 0.0747 |
| Superior parietal gyrus_L | 6.3621 ± 0.9300 | 6.9863 ± 1.3456 | 0.1035 |  | 0.1552 ± 0.0219 | 0.1635 ± 0.0153 | 0.0592 |  | 10.7585 ± 9.3336 | 16.9844 ± 11.3368 | **0.0115** |
| Medial orbital gyrus_L | 10.9312 ± 0.9998 | 11.2462 ± 0.6746 | 0.1170 |  | 0.2012 ± 0.0069 | 0.2051 ± 0.0058 | **0.0115** |  | 30.5831 ± 12.9806 | 35.9663 ± 13.5795 | 0.0838 |
| Pre-subgenual anterior cingulate gyrus_R | 5.1254 ± 2.3077 | 5.7895 ± 1.6053 | 0.2627 |  | 0.1423 ± 0.0307 | 0.1542 ± 0.0176 | **0.0432** |  | 28.7181 ± 19.0032 | 36.8428 ± 25.7074 | 0.1194 |

**Figure S7. Small-world topological properties (γ＞1 and λ≈1) of the gray matter network in SMA type 2 and type 3 based on KLDs method.**

**
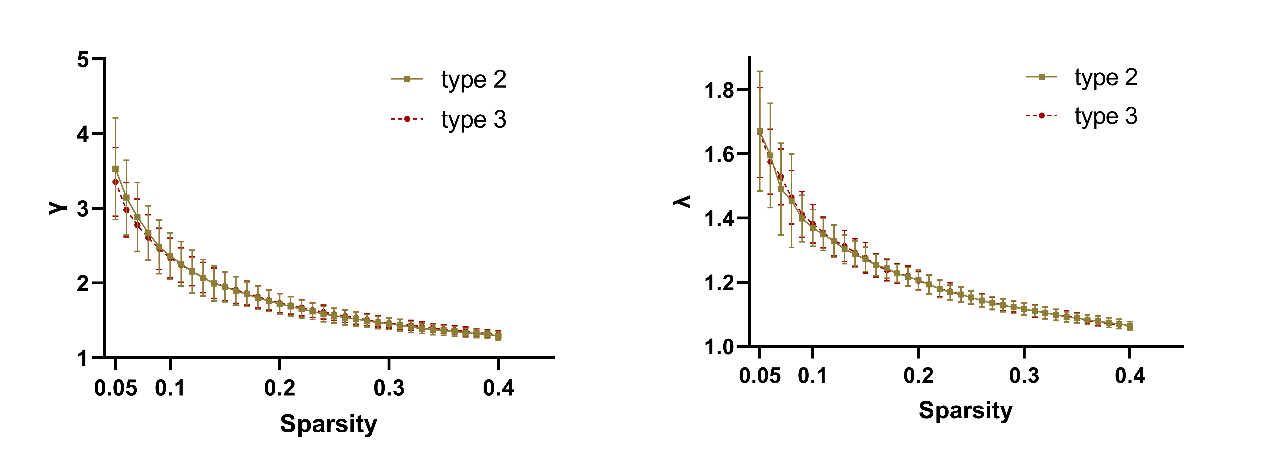
**

**Figure S8. Comparisons of global topological properties between SMA type 2 and type 3 based on KLDs method.**


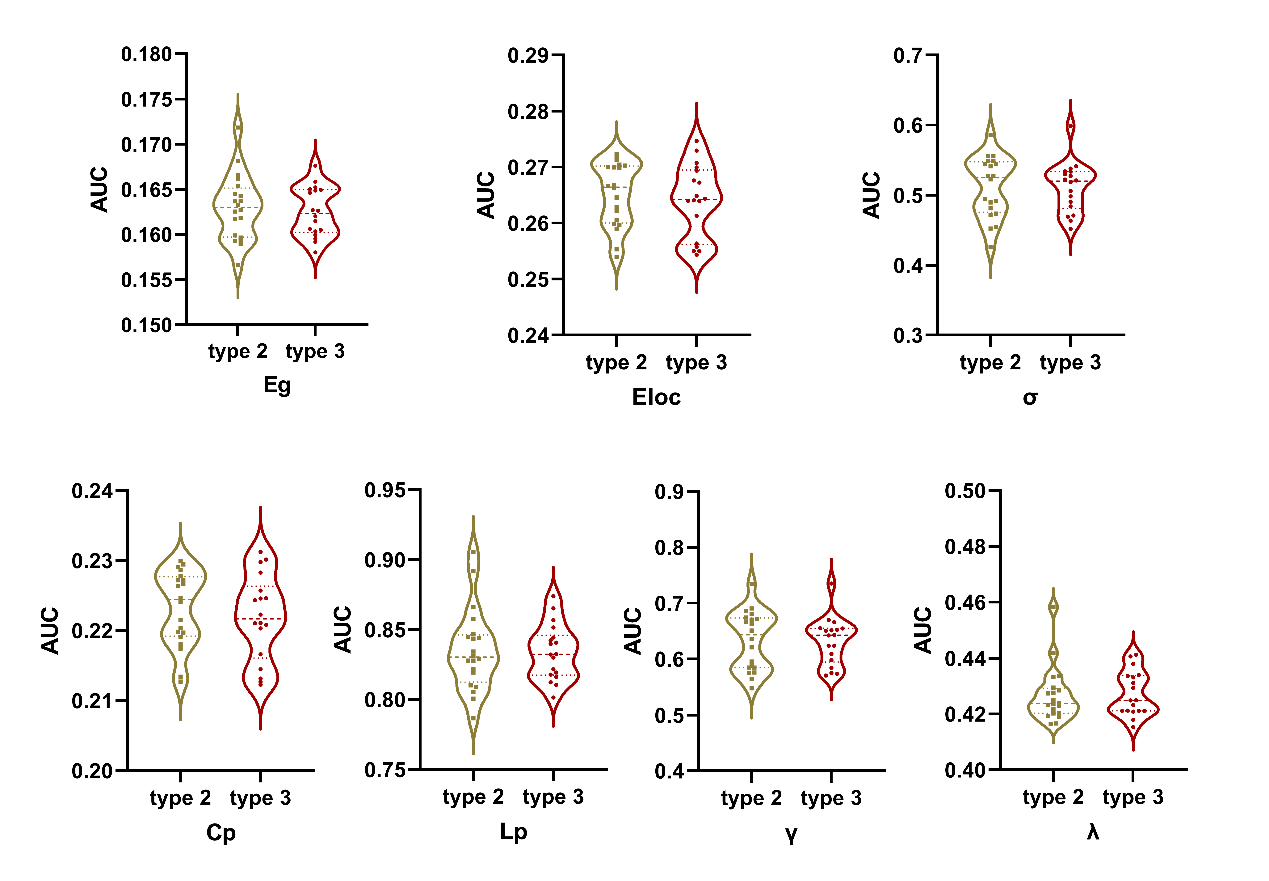

Supplement: Supplementary file 1 — Appendix S1. [file CNS-30-e14804-s001.docx]
